# Research on permeable pores in collapse column fillings with different gradation structures

**DOI:** 10.1038/s41598-022-11372-9

**Published:** 2022-05-07

**Authors:** Shuang Song, Tianjun Zhang, Hongyu Pan, Mingkun Pang, Xiufeng Zhang, Lei Zhang, Ruoyu Bao

**Affiliations:** 1 College of Safety Science and Engineering, Xi’an University of Science and Technology, Beilin District, No. 58, Yanta Middle Road, Xi’an city, 710054 Shaanxi Province China; 2 College of Science, Xi’an University of Science and Technology, Xi’an, 710054 China; 3 Information Institute, Ministry of Emergency Management of the PRC, Beijing, 100029 China

**Keywords:** Mineralogy, Fossil fuels

## Abstract

Particle loss is an important cause of water inrush catastrophes in collapsed columns. In order to study the relationship between the lost particles of different graded rock samples and the pore structure of the subsidence column filling, experiments were designed and the changes of the seepage parameters of graded rock samples during the particle migration process under different permeable water pressures P and axial loads F were determined. The results show that: (1) There will be obvious collapse, silting and particle loss behaviors in the sample during different loading processes, and the rock samples with gradation values of *n* = 0.3 and *n* = 0.5 are dominant; (2) The relationship between porosity *φ* and bearing pressure The exponential function can be used to fit the loads *F* well, and the porosity decreases with the increase of the bearing load. The water surging characteristics before and after 1.2 MPa are mainly in the turbulent water gushing stage, accompanied by instantaneous slurry. Possibility of splashing and indenter sliding; (3) After infiltration, the condition of the remaining skeleton rock samples in the cylinder generally shows a trend of first decreasing rapidly, then increasing slowly, and then decreasing; (4) The gradation value *n* of the sample and the bottom There is a good correlation between the damaged area and the mean value S of the maximum area of the top water inrush channel. The maximum area increase of the damaged area and the maximum area increase of the water inrush channel show an opposite trend. The permeable pores of the graded samples can be divided into There are three situations of digging and collapse, water inrush gap and scouring hole, and the pore seepage process can be divided into 4 stages of inoculation of water seepage, rapid adjustment, rapid scour and steady flow.

## Introduction

With increasing mining depth, the hydrogeological structural conditions encountered in underground excavation become increasingly complex and severe, and the threat to mining floor water damage becomes increasingly serious^1,2^. As the main manifestation of floor water damage, collapse column water inrush events are coal mine accidents with extremely negative effects in China^3,4^. Collapse column fillings are developed in karst water-rich areas and occur in a consolidated state. Under the combined action of coal mining and groundwater activities, the water pressure in Ordovician limestone water-rich aquifers is gradually transferred upwards, resulting in column filling collapse. Fine particles between objects migrate from the bottom to the top of the collapsed column^5^, resulting in the occurrence of a column pore structure, transformation of the water flow pattern, and finally water inrush disasters involving collapsed columns^6,7^. The pore structure characteristics and load-bearing permeability state of collapse column fillings constitute the key factors determining column collapse due to water inrush via water channels^8,9^. To better understand these problems and reduce the associated loss and impact, it is necessary to mitigate collapse attributed to particle migration^10–12^.

Collapse column filling is a closed structure with a low air permeability and suitable compactness, and the overlying rock layers mostly comprise fragments^13,14^. Many scholars have performed research on the permeability evaluation, cementation performance and compaction characteristics of collapse column fillings under particle migration. In permeability testing of rockfill samples, Bai^15^ regarded the collapse column as a plug shape and proposed the plug model to describe the permeability behaviour of coal seam floors containing collapse columns. Yao^16^ et al. employed a broken rock mass permeability test system satisfying particle migration to determine the permeability of broken rock samples at different ratios and analysed the porosity, permeability, and mass loss rate of broken rock samples over time. The relationship between seepage changes and the time-varying law of broken rock masses under different proportions was obtained. In terms of the cementation performance of fillings, Yu^17,18^ et al. applied an independently developed test device to conduct permeability tests of cemented and broken mudstone samples and studied different cementing agents, osmotic pressures, broken mudstone particle size distributions and initial porosities. As such, the change law of cemented broken mudstone permeability was established. Zhang^19,20^ et al. considered Talbol’s theory to prepare cemented and broken coal samples with different gradation structures, conducted steady-state tests of these broken coal samples with a triaxial permeameter and measured the sample porosity under different axial stresses^21,22^. The impact of the permeability k, non-Darcy flow factor β, critical instability value, cementation degree, pore structure and effective stress on the seepage stability of fractured coal were analysed. In terms of the compaction characteristics of rockfill samples, Zhang^23^ et al. implemented the MTS815.02 permeability test system based on the condensation and reshaping phenomena of broken rock blocks in collapse columns and applied the transient method for filling reshaping at different initial water contents. Various samples were tested, the influence of the confining pressure and initial water content on the sample permeability was analysed, and a permeability relationship for column fillings in different stress states was obtained. In regard to other aspects of pore permeability evaluation of collapse column fillings under particle migration, Feng^24^ used a seepage test system considering particle loss to conduct experimental research on the permeability characteristics and water inrush behaviour of red sandstone and examined the effect of different Talbol index n values on the quality. The influence of loss, porosity and permeability on the variation in the Talbol index n value was determined considering various permeability parameters. Wu^25^et al. measured the permeability characteristics of red sandstone, analysed the influence of mass loss on the porosity and permeability of broken rock samples under different Talbol index n values, and constructed a model of fractured rock samples based on the obtained experimental results. A non-Darcy seepage genetic algorithm was applied to obtain rock mass permeability characteristics^26–28^.

Collapse column filling mainly comprises broken rock fragments, and the pore structure is complex and variable. Under the combined action of mining activities and groundwater pressure, the influence of filling structural characteristics on permeable pores cannot be ignored, but experimental research in this area is lacking. Based on this, this article on the basis of the study, carried out does not match at the same level of the structure of the collapsing column fillings pore penetration test research, through the description of the experimental phenomenon and the related influence factors analysis, to study the variation characteristics of infiltration pore collapse column fillings, and then to collapse column water inrush disaster prevention, security enhancements of underground construction to provide the necessary theoretical and experimental support.

## Methods

### Test device and sample configuration

**Figure 1:**
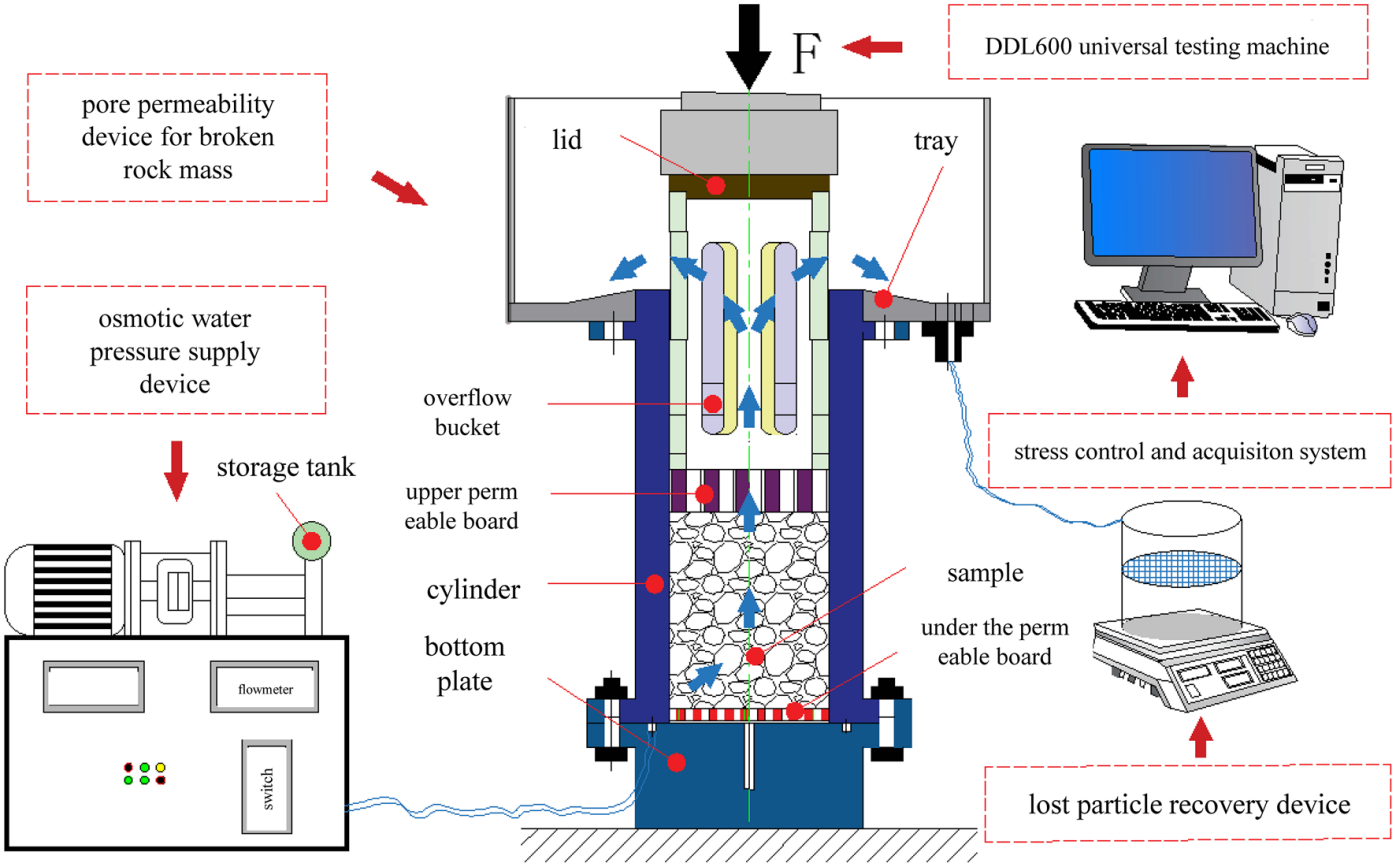
Test device.

This test relies on a self-designed and patented particle migration broken rock mass seepage test system. This modern test system combines computer information collection and digital measurement components. This test system can accurately measure the axial stress and axial displacement of rock samples and mainly includes a DDL600 electronic universal testing machine and osmotic water pressure supply. The system, broken rock mass pore penetration device, lost particle recovery device and stress control and collection system are shown in Fig. 1.

The mudstone tested was retrieved from the Yuwu Coal Mine in Shanxi Province. The hydrogeological conditions in this mine are complex. In the mining process, there exists the possibility of water inrush in the abnormal water-rich area of the coal seam floor with collapse columns. Targeting a rock block retrieved from the mine floor, a CP-330 hammer crusher was employed to crush the rock block, and a particle size sorting screen was adopted with a range from 0–25 mm. After the rock block fragments were sorted, 7 particle intervals were obtained, including 0–2.5 mm, 2.5–5 mm, 5–8 mm, 8–10 mm, 10–12 mm, 12–15 mm and 15–20 mm.

Rock block particles of various sizes easily form a high-dimensional parameter space, and the influence of the particle distribution on the test results is difficult to evaluate. To overcome the problem of the different dimensions of the rock block fragments, the Talbol gradation theory was considered to describe the particle size distribution in collapse column fillings, as follows:

1 $$P_{i} = \left\{ {\begin{array}{*{20}c} {\left[ {\left( {\frac{{d_{i + 1} }}{{D^{*} }}} \right)^{n} - \left( {\frac{{d_{i} }}{{D^{*} }}} \right)^{n} } \right] \times 100\% } & {\left( {i > 1} \right)} \\ {\left( {\frac{{d_{i} }}{{D^{*} }}} \right)^{n} \times 100\% } & {\left( {i = 1} \right)} \\ \end{array} } \right.$$

where Pi is the proportion of mudstone particles with a diameter smaller than di, d is the diameter of the mudstone particles, D* is the maximum diameter of the mudstone particles, and n is the Talbol power index. Among these variables, the effect of the particle size on the test results was reduced. The maximum diameter of the sample particles is 20 mm, which is 1/5 of the inner diameter of the cylinder. Each group of samples weighs 1800 g. According to Eq. (1), 4 groups of graded samples with gradation values n of 0.3, 0.5, 0.7 and 0.9 could be obtained in Table 1.

**Table 1:** Mass fraction of each particle size of the samples.

*d*_*i*_ (mm)	Mass fraction of each particle size *P*_*i*_ (%)			
	*n* = 0.3	*n* = 0.5	*n* = 0.7	*n* = 0.9
0–2.5	53.59	35.36	23.33	15.39
2.5–5	12.39	14.64	14.57	13.33
5–8	9.99	13.25	14.76	15.12
8–10	5.26	7.47	8.90	9.75
10–12	4.57	6.75	8.38	9.56
12–15	5.94	9.14	11.82	14.04
15–20	8.27	13.40	18.24	22.81

The mass distribution of each particle size at the different ratios is summarized in Table 2.

**Table 2:** Mass distribution of each particle size of the samples.

*d*_*i*_ (mm)	Particle size (g)			
	*n* = 0.3	*n* = 0.5	*n* = 0.7	*n* = 0.9
0–2.5	964.6	636.4	419.9	277.0
2.5–5	223.0	263.6	262.2	239.9
5–8	179.8	238.4	265.7	272.2
8–10	94.7	134.4	160.2	175.5
10–12	82.2	121.5	150.8	172.0
12–15	106.9	164.6	212.8	252.8
15–20	148.8	241.2	328.3	410.6

According to Table 2, a high-precision electronic scale with a range from 0–3000 g, a division value of 0.01 g, and an error range of ± 0.03 g was adopted to weigh mudstone samples of different particle sizes. Mixed-particle size samples with gradation values n of 0.3, 0.5, 0.7 and 0.9 were obtained after proportioning.

### Test procedure

To simulate the pore penetration process in collapse column fillings at different burial depths during mining activities, we describe pore penetration test phenomena at various axial load levels and analyse migrating particles, pressure load, particle size ratio, rock block skeleton and permeable pores. The relationship between these parameters reveals the change characteristics of penetrating pores in collapse column fillings. Based on the prepared sets of mixed-particle size samples with Talbol power exponents n of 0.3, 0.5, 0.7 and 0.9, the improved particle migration and seepage test system applied the constant-load restraint method to carry out the test.

The water conductivity of a collapse column is directly related to the permeable pores in the filling material. Many scholars have researched the percolation evolution law of collapse columns. However, due to the limitations of test equipment and test conditions, there are relatively few studies on the characteristics of permeable pore changes in collapse column fillings with different gradation structures. Based on this lack, in this paper, pore permeability tests are performed on collapse column fillings with different gradation structures, the pore permeability process is simulated in collapse column fillings at various burial depths during mining activities, and pore permeability test phenomena are described at various axial load levels. The relationship among migrating particles, pressure load, particle size ratio and rock block skeleton and permeable pores was determined, and the permeable pore change process in rockfill samples was characterized.

### Pore permeability test phenomena among the rock samples with different grades

**Figure 2:**
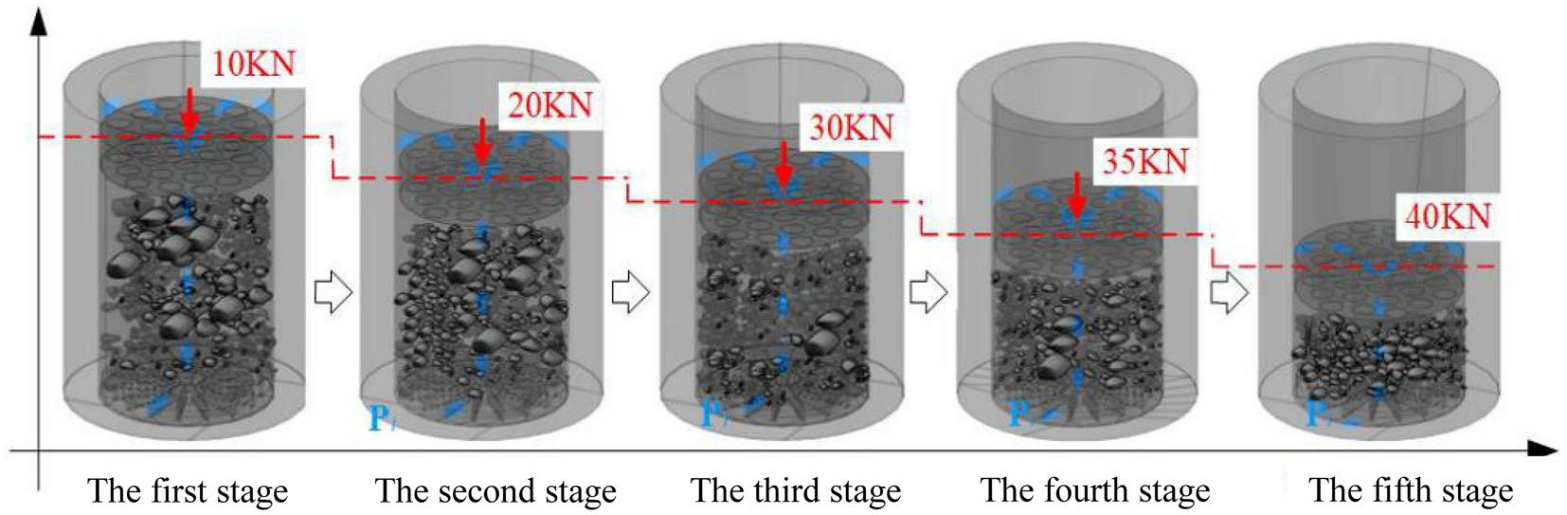
Pore permeability test process of the samples at the various load stages.

Among the multiple sets of graded mixed rock samples of different gradation structures with Talbol power exponents n of 0.3, 0.5, 0.7, and 0.9, pore permeability tests were carried out. The pore permeability test process applied to the samples at the various load stages is shown in Fig. 2.

Figure 2 shows that the filling sample is continuously compacted under the action of 5 axial load levels, and the sample height is reduced in a stepwise manner during the loading process. Under the effect of the pore water pressure, a specific water channel is formed in the sample.

**Figure 3:**
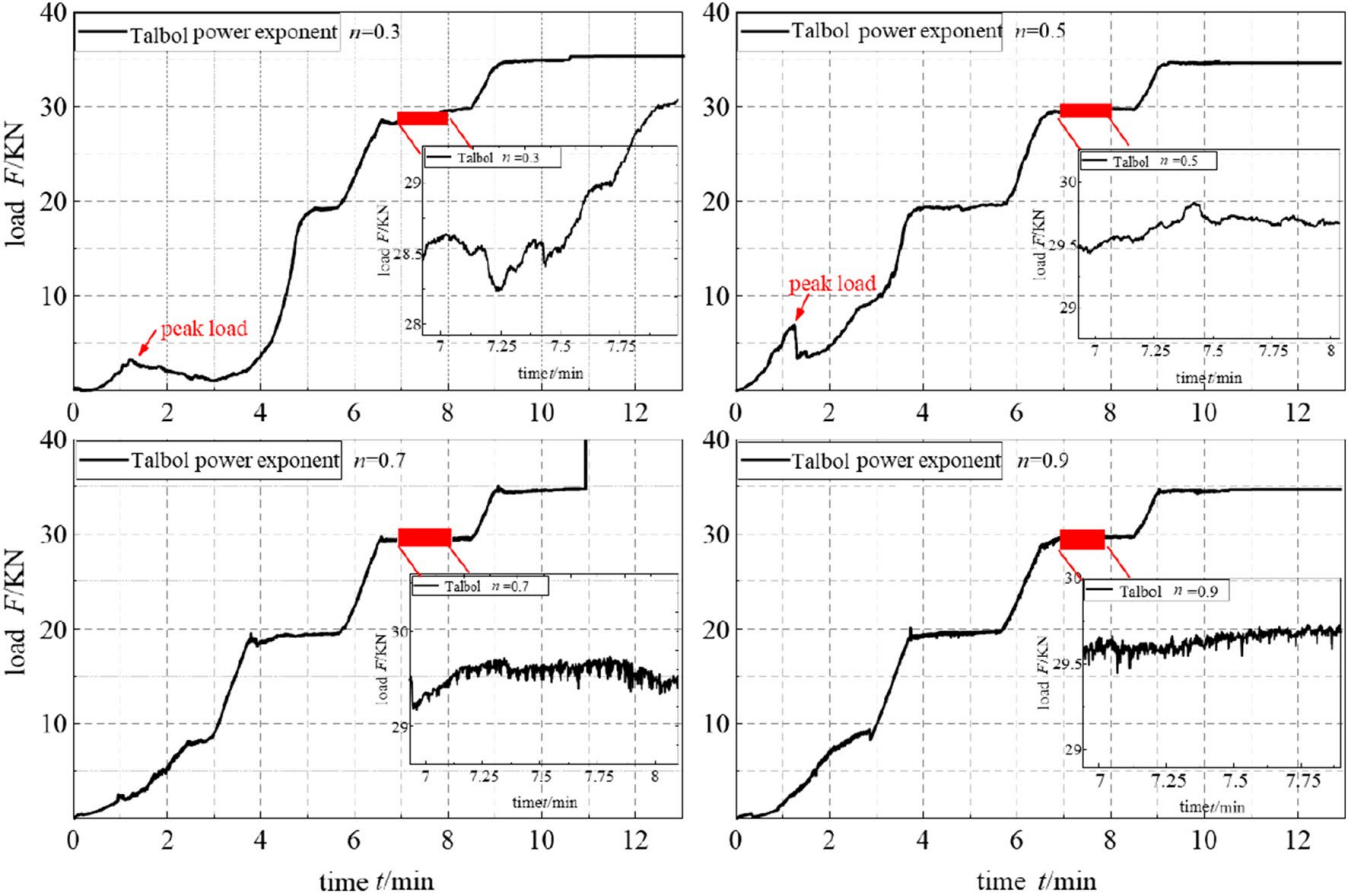
Relationship between the sample time and load in sample testing.

Combining the stress control and acquisition systems, the relationship between the sample time and load changes for the different gradation structures can be obtained, as shown in Fig. 3.

1. The axial load F and sample time t at each level follow a step-like distribution. Upon extension of the infiltration stage, the load eventually stabilizes, and there is a peak compaction load of approximately 35.3 N. After the second load level of 20 kN, the samples of different grades exhibit similar change trends.
2. Large-scale load fluctuation generally occurs at the middle and late stages of the first load level of 10 kN. The filling samples with gradation values n = 0.3 and n = 0.5 attain a notable performance, and load peaks of 1.293 and 6.747 kN, respectively, are observed. The load F at this stage increases with increasing gradation value n. Even though the samples with gradation values n = 0.7 and n = 0.9 still exhibit a certain load fluctuation behaviour, it is not notable.
3. The samples with gradation values n = 0.3 and n = 0.5 exhibit obvious load peak-induced collapse behaviour before and after 68 s, respectively, while the samples with gradation values n = 0.7 and n = 0.9 basically indicate no such characteristics. The latter samples exhibit a gradual progression over time and a rising change trend. Based on the partially enlarged view of the third load level of 30 kN, it can be observed that with increasing gradation value n, the load gradually stabilizes. Although certain load fluctuations remain, they are not significant and exhibit a gradually increasing similarity.

The above analysis results indicate that the samples at all levels achieve a certain hydraulic bearing capacity at the initial stage of loading. With increasing seepage pressure, the samples with gradation values of n = 0.3 and n = 0.5 are more likely to lose fine particles. Collapse and plugging phenomena occur, which are generally observed in the interval from 10–15 s after the first-level peak load. These phenomena cause rapid load fluctuation and adjustment among the filling samples with gradation values n = 0.7 and n = 0.9 attributable to the large internal void spaces. There are many fine particles in the pores, resulting in particle storage and migration behaviours, the hydraulic carrying capacity is relatively good, and the load fluctuation characteristics in the entire infiltration process are similar. At the 4th and 5th load levels of 40 and 50 kN, respectively, the load value of the sample with gradation value n = 0.9 was the closest, which indicates that the rock sample was basically compacted at this stage and that a seepage channel had formed.

### Conversion process of permeate flow at the initial stage

Choosing the filling sample with a Talbol power index n = 0.5 as an example, permeate was collected at the different osmotic pressure levels and load stages. The degree of turbidity of the permeate at the fifth load stage exhibited a change trend of slightly turbid → turbid → more turbid → clear → slightly clear, and the performance of the samples with gradation values n = 0.3 and n = 0.5 was more notable. This indicates that the internal structure of the graded sample resulted in poor stability, the water blocking ability was weak, and fine particles easily migrated. However, the permeate of the samples with gradation values of n = 0.7 and n = 0.9 was relatively turbid, indicating a certain hydraulic carrying capacity. At the middle and late stages of infiltration, choosing the filling sample with a Talbol power exponent n = 0.5 as an example, under the action of the first load level of 10 kN, the water flow conversion process in the water inrush channel area at the top of the filling sample and the permeate state were determined.

**Figure 4:**
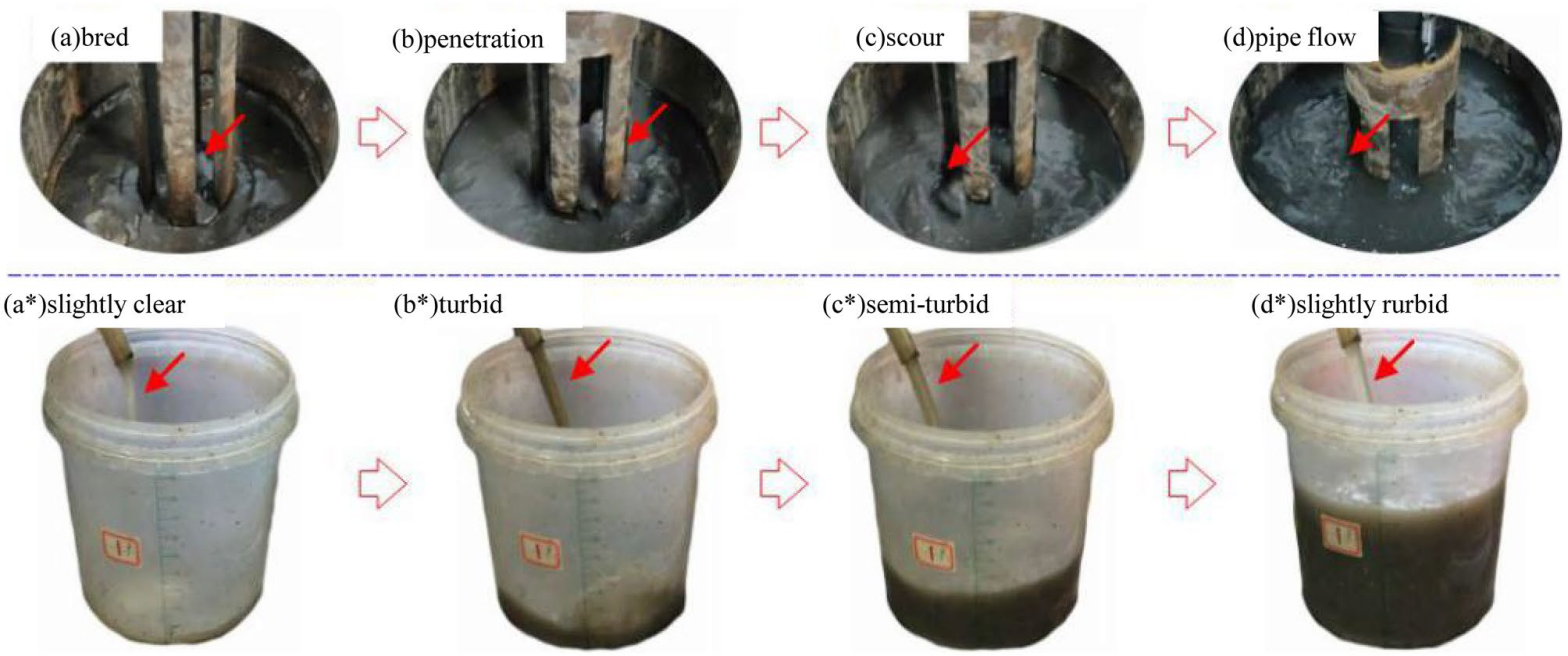
Water flow conversion process in the water inrush channel area at the top of the sample.

Figure 4 reveals that under the action of the pore water pressure, after a short period of loading and incubation of the filling sample (a), the sample gradually deteriorated due to erosion under a 0.8-MPa water pressure, and the internal pores and framework structure expanded. During expansion, water flowed through the weak area of the filling sample and was discharged, and the permeate was slightly clear (a*). After infiltration (b), under the action of an osmotic water pressure of 1.2 MPa, the permeate rapidly became turbid (b*), infiltrated fine particles were entrained, and the water flow into the tray exhibited obvious fluctuation characteristics. When a potential water channel had formed, the continuous softening and scouring effects of the water flow (c) could cause the internal pores in the filling to locally collapse, thus reducing the osmotic pressure, and the permeate increasingly became semiturbid (c*). After the water inrush channel was fully connected or had matured, the water flow pattern changed to a relatively stable pipe flow water inrush pattern (d). At this stage, the water flow remained relatively stable, without obvious fluctuations (d*), and the particle migration behaviour was reduced.

## Discussion

### Time-varying law of the migrating particles under the different osmotic pressures

**Figure 5:**
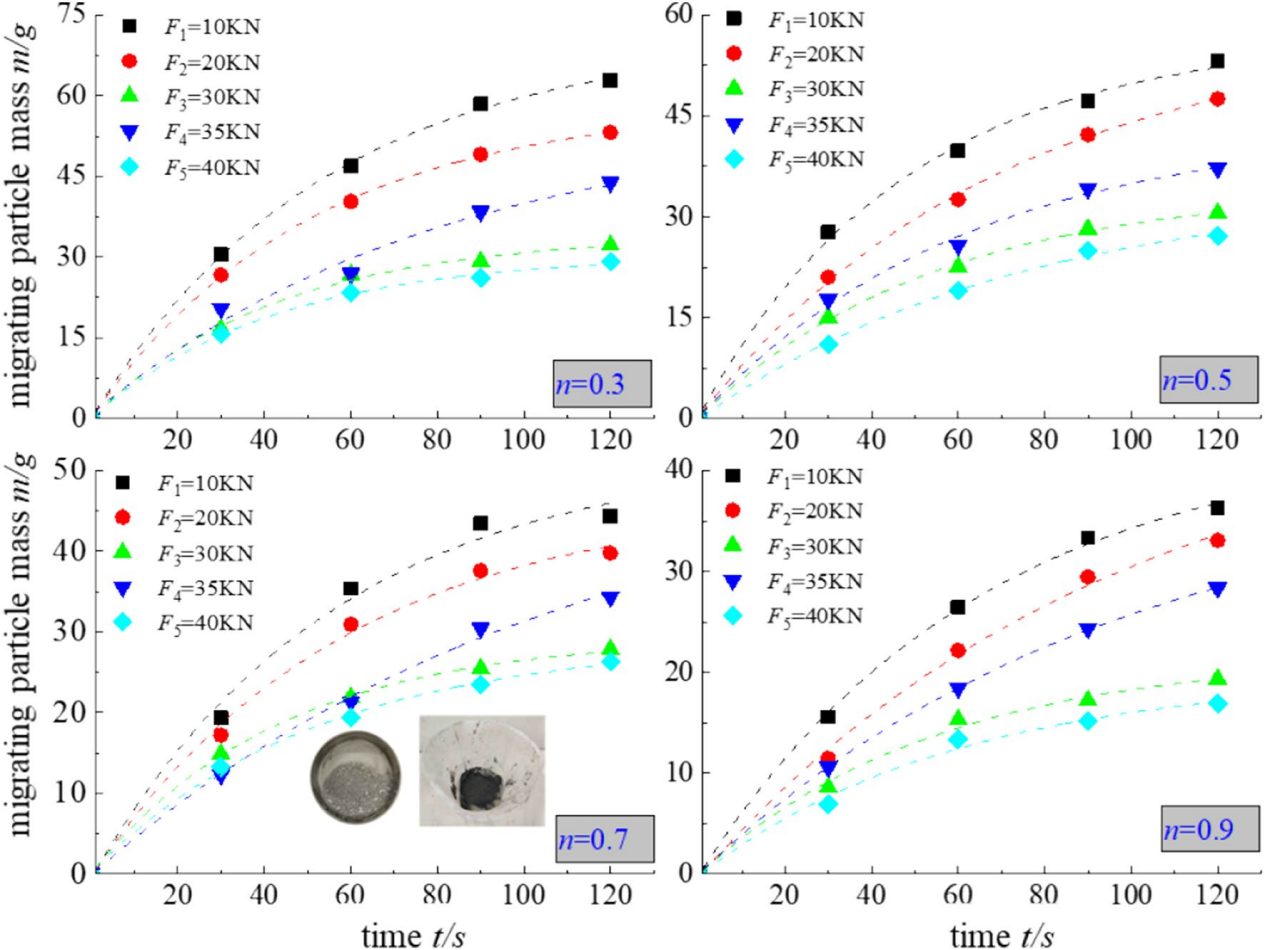
Mass of the migrating particles during the different time periods under the various loads.

When performing the pore penetration tests of the samples at all load levels, the load value was fixed, migrating particles were collected at a rate of 30 s/time under the 4 single-stage osmotic pressures, and the remaining particles were processed in a YED/HS-150 constant-temperature and constant-humidity test box at 140 °C for 3 h. After moisture stabilization, the relationship between migrating particles and time under the different osmotic pressures at the various load levels is shown in Fig. 5.

Under the different osmotic pressures at the various load levels, the mass m of the migrating particles decreased with increasing Talbol power exponent n, and the total mass m of the migrating particles was negatively correlated with the load F. The samples with gradation values n = 0.3 and n = 0.5 at the 10-kN load level and 1.6-MPa osmotic pressure produced more migrating particles, at 62.89 and 53.22 g, respectively, while the samples with gradation values n = 0.7 and n = 0.9 produced fewer migrating particles, at only 44.34 and 36.34 g, respectively. With increasing osmotic pressure, the quality of the migrating particles gradually stabilized. Under the 1.6-MPa osmotic pressure, in the 20–30 kN load interval, a large difference in migrating particles occurred, while in the 35–40 kN load interval, there was a small difference in migrating particles, at 15.36, 14.73, 11.67, and 11.45 g and 8.1, 6.64, 6.51, and 5.36 g, respectively. This indicates that the water inrush channel within the 20–30 kN load interval occurred at the stage of gradual deterioration and had nearly formed, while the pore channel in the 35–40 kN load interval occurred in a relatively stable state. The fitting relationship between the mass of migrating particles and time under the various osmotic water pressures is summarized in Table 3.

**Table 3:** Fitting relationship between the quality of the migrating particles and time under the various osmotic pressures.

Talbol power exponent	Load *F*/kN	Fitting equation	Fitting coefficient *R*^2^
*n* = 0.3	10	*m* = 70.96–70.95e^−0.019*t*^	0.9989
	20	*m* = 58.59–58.52e^−0.02*t*^	0.9995
	30	*m* = 34.06–34.15e^−0.024*t*^	0.9943
	35	*m* = 55.88–55.17e^−0.012*t*^	0.9737
	40	*m* = 30.44–30.4e^−0.024*t*^	0.9971
*n* = 0.5	10	*m* = 56.95–56.65e^−0.021*t*^	0.9955
	20	*m* = 57.81–57.64e^−0.014*t*^	0.9982
	30	*m* = 34.3–34.24e^−0.019*t*^	0.9985
	35	*m* = 43.64–43.45e^−0.016*t*^	0.9930
	40	*m* = 34.36–34.5e^−0.014*t*^	0.9961
*n* = 0.7	10	*m* = 52.14–52.73e^−0.018*t*^	0.9829
	20	*m* = 46.61–47e^−0.017*t*^	0.9911
	30	*m* = 29.47–29.42e^−0.023*t*^	0.9993
	35	*m* = 52.96–53.08e^−0.009*t*^	0.9947
	40	*m* = 28.8–28.66e^−0.019*t*^	0.9966
*n* = 0.9	10	*m* = 32.62–32.53e^−0.02*t*^	0.9981
	20	*m* = 47.52–47.87e^−0.01*t*^	0.9940
	30	*m* = 21.76–21.89e^−0.018*t*^	0.9907
	35	*m* = 40–40e^−0.01*t*^	0.9998
	40	*m* = 19.73–19.92e^−0.017*t*^	0.9854

Table 3 and Fig. 5 show that the mass of the migrating particles m and time t under each osmotic water pressure can be fitted with an exponential function *m*=*b*+*e*^−*t*^, and the fitting equation achieves a good correlation with the experimental data.

### Variation relationship of the permeable pores under the various pressure loads

According to the design dimensions of the various components of the broken rock mass pore penetration device, the initial accumulation height of the different graded samples in the lower cylinder at each load stage can be directly calculated:

2 $$h_{0} = H_{5} + H_{6} - H_{1} - H_{2} - H_{3} - H_{4}$$

In the above equation, H_6_ is the height of the indenter at the different load stages of the various samples, which can be directly measured with a ruler. H1–H5 are determined during design, and h0 can be directly calculated from H_6_, as follows:

3 $$h_{0} = H_{6} - 75$$

The initial indenter heights of the samples at each level during loading are provided in Table 4.

**Table 4:** Initial indenter height at the different load stages.

Load level	Load *F*/kN	Pressure *P*/MPa	Compression displacement *H*_6_/mm			
			*n* = 0.3	*n* = 0.5	*n* = 0.7	*n* = 0.9
1	10	1.27	140	149	165	170
2	20	2.55	95	102	149	159
3	30	3.82	76	81	119	131
4	35	4.46	64	69	94	101
5	40	5.1	56	61	69	74

After filling the sample loading into the cylinder, the height of the graded samples with Talbol power index n values of 0.3, 0.5, 0.7 and 0.9 can be measured, at h1 = 18.9 cm, h2 = 19.4 cm, h3 = 19.8 cm and h4 = 20.5 cm, respectively, and the initial porosity corresponding to each load level is φ1 = 0.524, φ2 = 0.536, φ3 = 0.545 and φ4 = 0.561. Then, the sample porosity after loading is:

4 $$\varphi_{0} = 1 - \frac{M}{{\pi a^{2} h_{0} \rho }}_{m}$$

where M is the mass of the broken rock sample, kg; a is the inner diameter of the cylinder, m; h0 is the height of the sample, m; and ρm is the mass density of the broken rock sample, kg/m3.

In the process of particle loss, the porosity of the samples at the different levels under the various seepage pressures at the same stage is:

5 $$\varphi_{i} { = }\varphi_{0} { + }\frac{1}{{\pi {\text{a}}^{2} h\rho_{m} }}\left( {\Delta m_{1} + \Delta m_{2} + \cdot \cdot \cdot + \Delta m_{i} } \right)$$

In the above equation, mi is the particle loss mass of the broken rock sample, kg.

**Figure 6:**
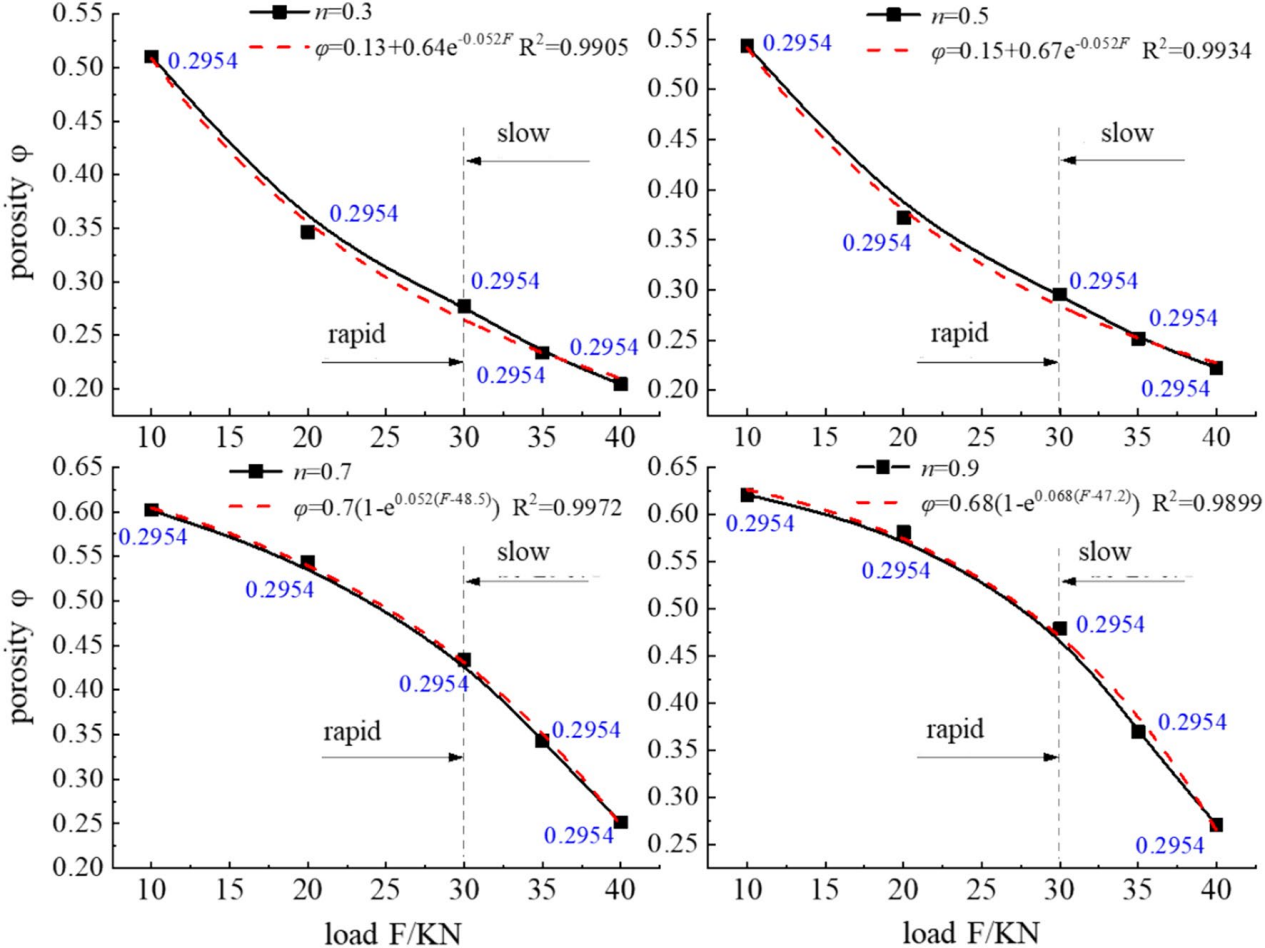
Relationship between the porosity changes under the different loads.

To further characterize the relationship between the load F and porosity φ of the different graded samples, an exponential function is applied to fit the relationship between the load F and porosity φ, as shown in Fig. 6.

Figure 6 shows that during the entire pore penetration process, the porosity decreases with increasing load, and the test data and the fitting equation between the porosity φ and pressure load F attain a good correlation. The change process of the porosity can be divided into two stages, namely, rapid and slow change stages, with 30 kN as the demarcation point. At the rapid porosity change stage, the porosity difference between the samples with gradation values n = 0.3 and n = 0.5 greatly decreased, at 0.1641 and 0.1714, respectively, which occurred in the 10–20 kN interval. In contrast, the porosity reduction in the samples with gradation values n = 0.7 and 0.9 was relatively limited, but a large reduction occurred in the 20–30 kN interval, at 0.1094 and 0.1022, respectively, as reflected by the hydraulic carrying capacity. At the slow porosity change stage, the porosity reduction in the samples with gradation values n = 0.3 and n = 0.5 was much smaller than that in the samples with gradation values n = 0.7 and n = 0.9. This indicates that the seepage channels in the samples with gradation values n = 0.3 and n = 0.5 had basically formed, while the samples with gradation values n = 0.7 and n = 0.9 still contained a certain pore expansion space. Moreover, the pores occurred in a state of gradual deterioration.

### Effect of the particle size ratio on the penetration pores of the sample

After infiltration, the remaining rock skeleton samples in the cylinder were processed to remove the remaining water followed by screening and weighing. The total masses of the remaining rock skeleton samples with gradation values n of 0.3, 0.5, 0.7 and 0.9 were 891.97, 973.42, 1102.56 and 1187.41 g, respectively. The distribution in each particle size interval of the remaining rock skeleton samples in the cylinder is provided in Table 5.

**Table 5:** Distribution in each particle size interval of the remaining rock skeleton samples in the cylinder.

*d*_*i*_ (mm)	Mass of each particle size (g)			
	*n* = 0.3	*n* = 0.5	*n* = 0.7	*n* = 0.9
0–2.5	243.91	295.01	362.18	431.18
2.5–5	135.45	141.26	158.16	213.6
5–8	101.28	96.36	103.11	99.27
8–10	80.44	89.59	87.57	94.05
10–12	77.37	64.42	98.93	65.15
12–15	85.62	94.67	120.18	128.66
15–20	74.08	117.79	111.88	116.87
> 20	93.82	74.32	60.55	38.63

Table 5 indicates that after infiltration, the proportion of each particle size interval of the remaining rock samples in the cylinder generally exhibits a trend of an initial rapid decrease followed by a slow increase and finally a decrease again. The particle size in the 8–10 mm interval is chosen as the boundary. There are a small number of consolidated rock fragments with a particle size larger than 20 mm. Among the samples with gradation values of n = 0.3 and n = 0.5, the proportions of the 0–2.5 mm and > 20 mm particle size ranges are 27.35% and 30.31%, respectively, and 10.52% and 7.63%, respectively, while among the samples with gradation values of n = 0.7 and n = 0.9, the proportions of the 0–2.5 mm and > 20 mm particle size ranges are 32.85% and 36.73%, respectively, and 5.49% and 3.29%, respectively. The proportions of the 0–2.5 mm and > 20 mm particle size ranges differ between the samples with varying gradation values n. The particle size range shows a diametrically opposite trend. The occurrence of this phenomenon indicates that the samples with gradation values n = 0.3 and n = 0.5 attain a better compaction and cementation performance between the particles after water seepage and loading. A certain amount of fine particles is bonded and recombined. The fragmentation degree of the samples with gradation values n = 0.7 and n = 0.9 is notable.

**Figure 7:**
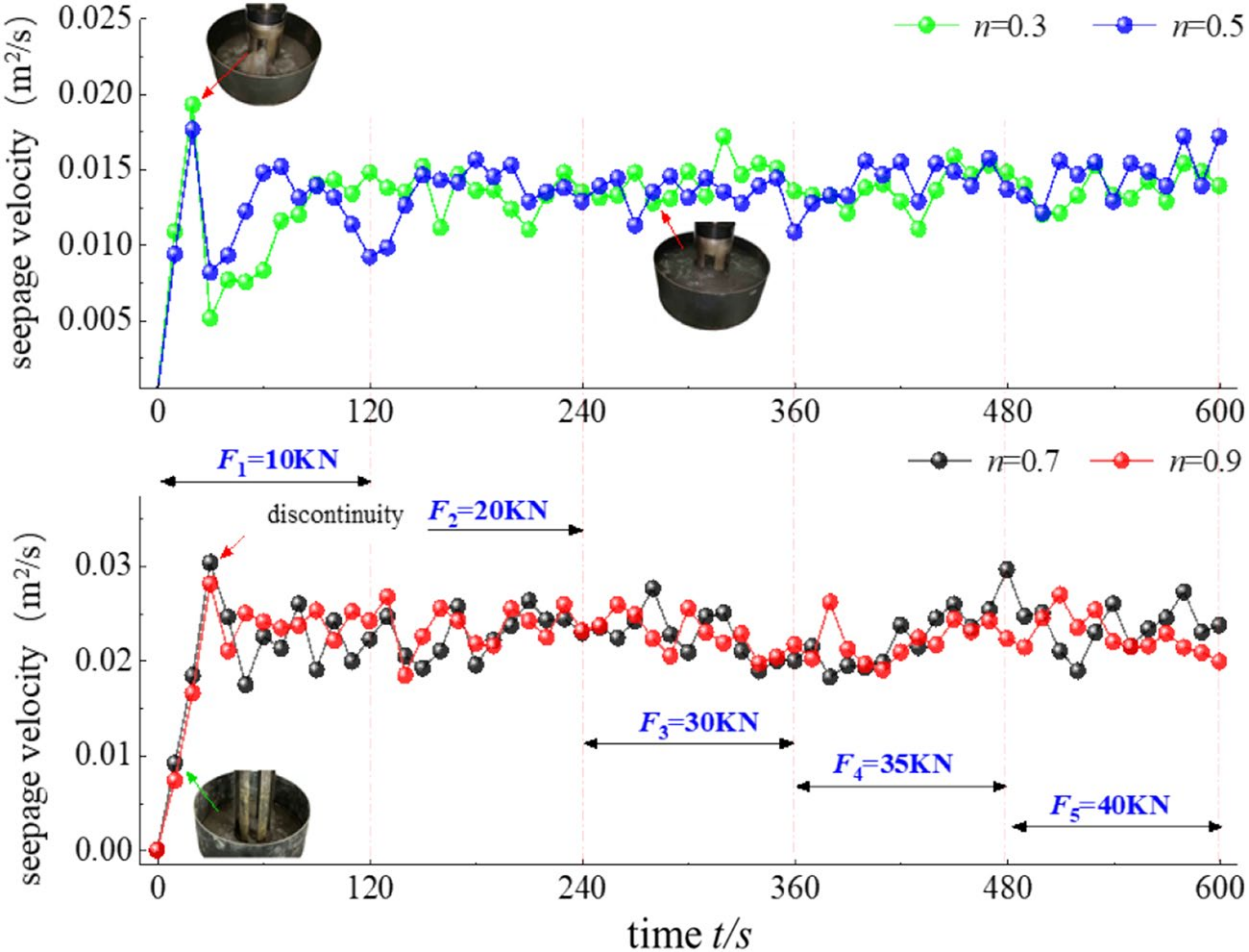
Relationship between the flow rate and time.

During the test, the water seepage quality is determined in real time with an electronic scale, and a time series of the water quality is collected at 10-s intervals. The difference method is applied to obtain the water inrush rate at a certain moment according to the quality change.

6 $$v_{t = i} = \frac{{m_{i} - m_{i - 1} }}{{\rho_{w} \pi a^{2} \Delta t}}$$

where m is the quality of the effluent, kg; ρw is the mass density of water, kg/m3; a is the inner radius of the cylinder, m; and Δt is the sampling time interval, s. The relationship between the flow velocity and time at the different permeation stages is shown in Fig. 7.

The relationship between the change in seepage velocity and time at all load levels is generally very similar. At the first stage of the 10-kN load level, there is a sudden change point, and the water flow fluctuates greatly. The performance of the samples with gradation values n = 0.3 and n = 0.5 is very obvious. The maximum seepage velocities are 0.0193 and 0.0176 m2/s, respectively. The water inrush behaviour of the samples with gradation values of n = 0.7 and n = 0.9 is observed later, and the maximum seepage velocities are 0.0303 and 0.0281 m2/s, respectively. This indicates that the seepage velocity is related to the internal pore structure of the graded samples. The lower the gradation value n is, the easier it is to expose fine particles, which migrate sooner.

**Figure 8:**
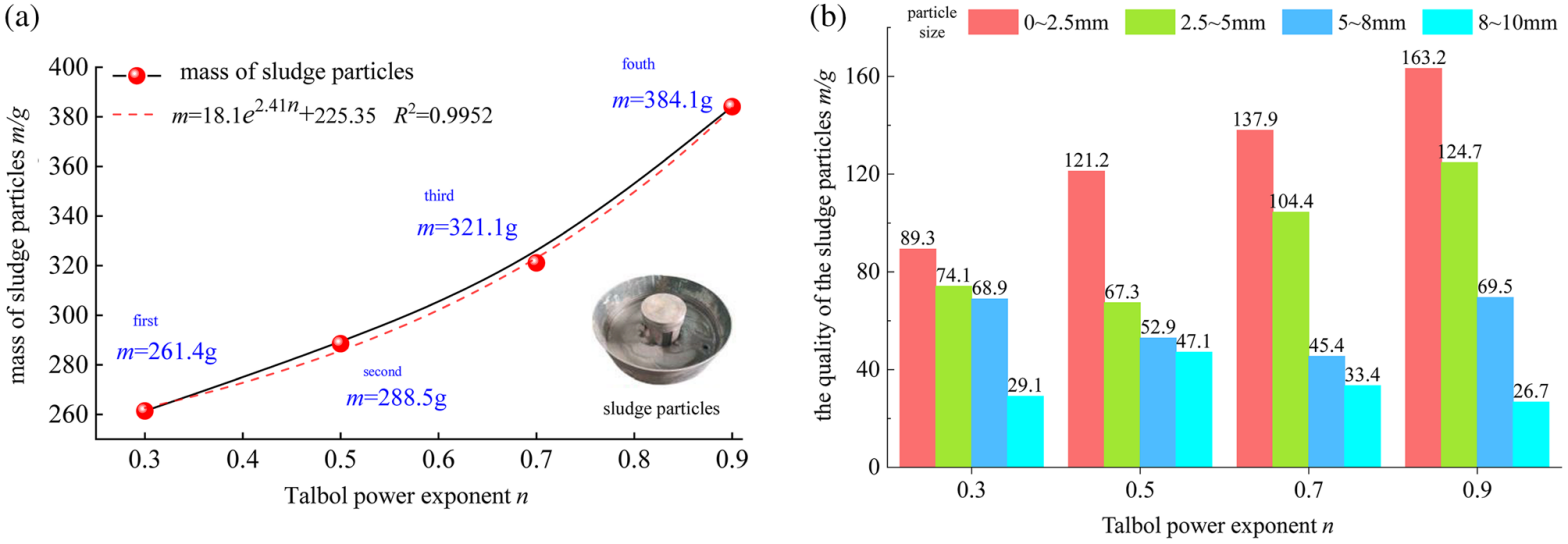
Silt particles of the samples at the different levels.

After loading and infiltration, the mass of silt particles on the tray was 261.4, 288.5, 321.1 and 384.1 g. The relationship between the gradation value n and mass of silt particles and the distribution of the various particle size intervals are shown in Fig. 8.

The mass m of silt particles increases with increasing value of the Talbol power exponent n, and an exponential function can be employed to suitably fit the gradation value n and mass m of silt particles. In terms of the distribution of the silt particles of the samples with different grades, most of the particles occur in the range from 0–2.5 mm. In this interval, the mass of the silt particles of the samples with different grades is 89.3, 121.2, 137.9 and 163.2 g, with few particles in the 8–10 mm interval. The particle size is randomly distributed in the 2.5–8 mm interval, which indicates that the samples with gradation values ​of n = 0.7 and n = 0.9 likely produce more fine particles. Large particles reduce the hydraulic carrying capacity of the graded sample.

### Effect of the particle size ratio on the pore channels

**Figure 9:**
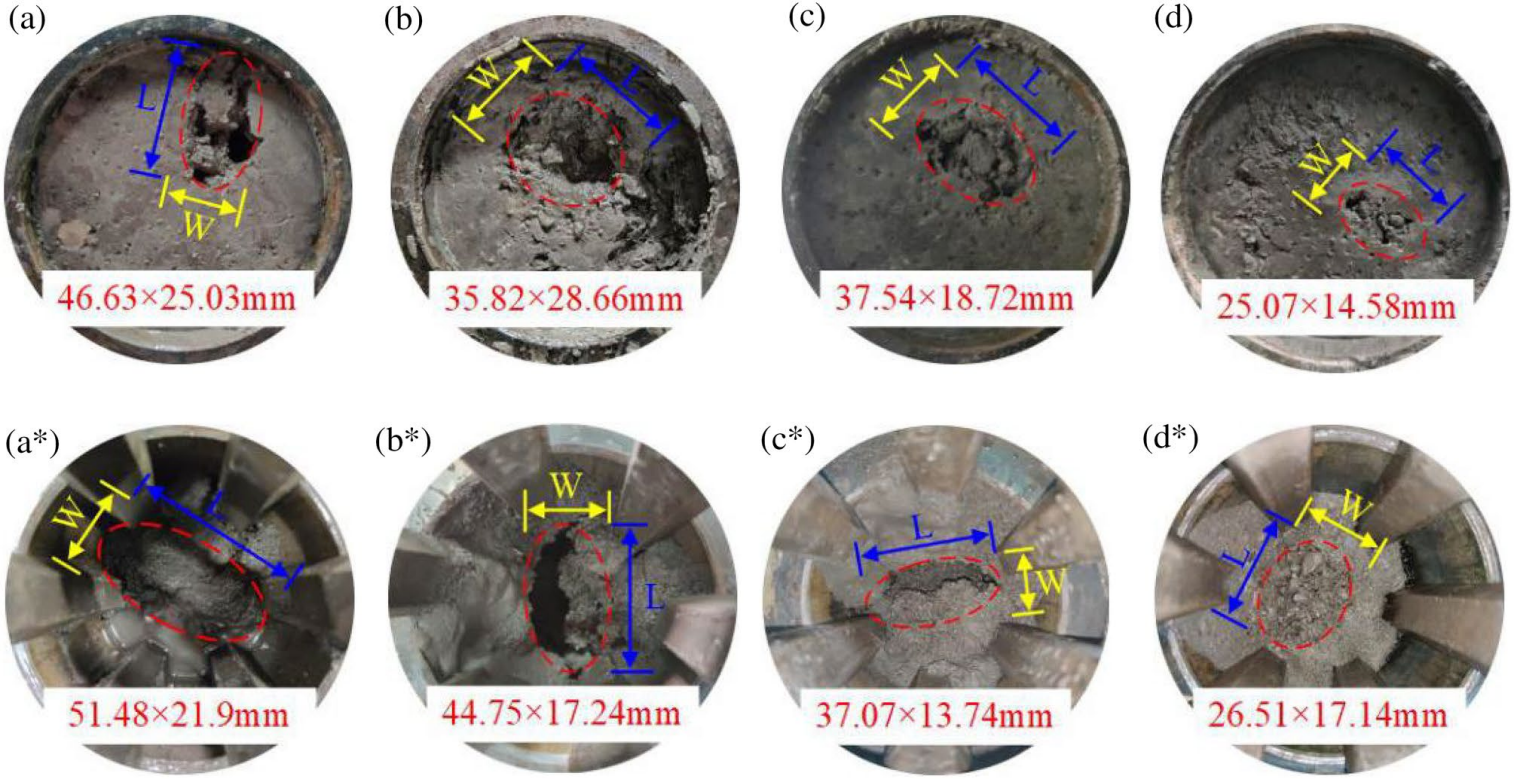
Bottom failure area and top water inrush channel of the samples at each level.

After infiltration, the bottom failure area and top water inrush channel of the filling samples at each level are shown in Fig. 9.

Figure 9 shows that the higher the particle size gradation value n is, the higher the hydraulic carrying capacity. The flow of water through the gaps and spaces in the sample facilitates the accumulation and loss of fine particles, which directly drives the increase in porosity and the formation of perforated throat corridors. Relatively unstable areas are prone to form larger pore structures (b).

With the help of rulers and software measurement tools to determine 10 actual measurement statistics, the maximum average size of the bottom failure area of the filling samples with gradation values n of 0.3, 0.5, 0.7 and 0.9 can be obtained, numbered M-1, M-2, M-3, and M-4, respectively (Table 6).

**Table 6:** Size of the damaged area at the bottom of the samples at all levels.

No	Talbol power exponent *n*	Maximum mean size		
		length L/mm	width W/mm	area S/mm^2^
M-1	0.3	46.63	25.03	1167.15
M-2	0.5	35.82	28.66	1026.60
M-3	0.7	37.54	18.72	702.75
M-4	0.9	25.07	14.58	365.52

Numbered as N-1, N-2, N-3 and N-4, the size of the water inrush channel at the top of the filling samples at each level is summarized in Table 7.

**Table 7:** Size of the water inrush channel at the top of the samples at all levels.

No	Talbol power exponent *n*	maximum mean size		
		length L/mm	width W/mm	area S/mm^2^
N-1	0.3	51.48	21.9	1127.4
N-2	0.5	44.75	17.24	771.49
N-3	0.7	37.07	13.74	509.34
N-4	0.9	26.51	17.14	454.38

**Figure 10:**
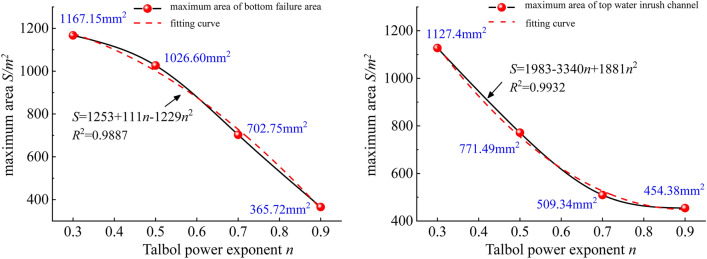
Relationship between the maximum area of the top water inrush channel and bottom failure area of the different graded samples.

To further characterize the relationship between the rockfill sample gradation value n, bottom failure area and maximum area S of the top water inrush channel, a second-order function is employed for fitting, as shown in Fig. 10.

According to the analysis of Fig. 10 and Tables 6 and 7, there exists a good correlation between the filling sample gradation value n and the bottom failure area and maximum area S of the top water inrush channel. The maximum increase in the bottom failure area is related to water inrush. The increase in the maximum area of the top water inrush channel exhibits the opposite trend. The maximum area of the M-1 damage zone is 1167.15 mm2, the minimum area of the M-4 damage zone is 367.72 mm2, the maximum area of the N-1 damage zone is 1127.4 mm2, and the minimum area of the N-4 damage zone is 454.38 mm2. There exists a certain intersection point between the damage area and water inrush channel, which mostly occurs among the filling rock samples with gradation values n = 0.7 and n = 0.9, thus indicating that there exists a certain interlocking and water-resistant interval in the gradation structure. This structure can withstand a certain amount of water erosion without collapsing.

### Variation characteristics of the pore channels in the collapse column fillings

**Figure 11:**
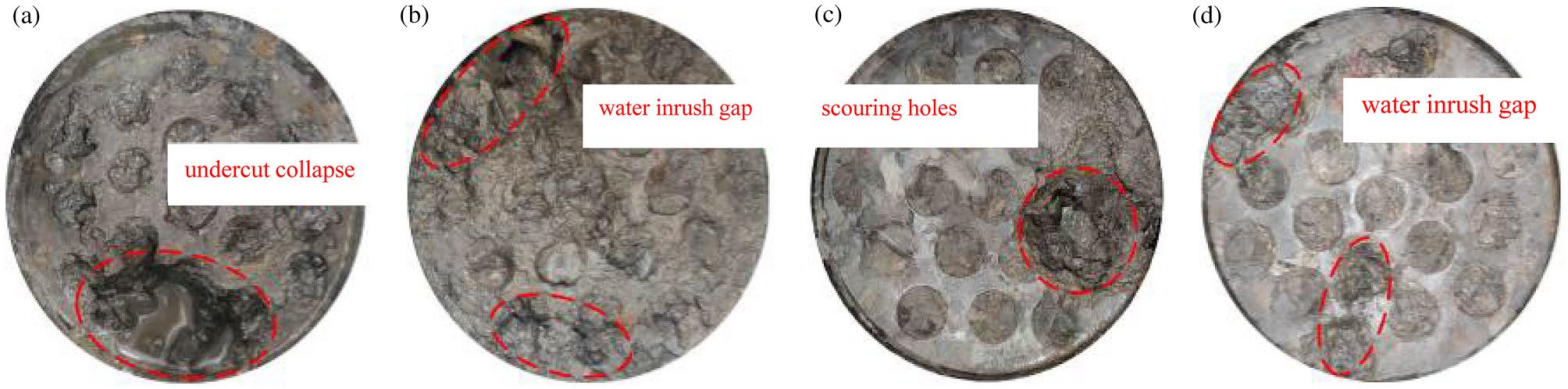
Pore channel conditions of each grade of sample after infiltration.

The test results revealed that the permeable pores in the different graded samples can be divided into three categories: digging collapse pores, water inrush crevices and flushing holes. The permeable pores in the graded samples after infiltration are shown in Fig. 11.

Figure 11 shows that the permeable pores in the filling samples are mostly located on both sides of the cylinder wall, revealing an irregular shape with a number of small tube-like holes. The filling samples with gradation values of n = 0.3 and n = 0.5 contain more obvious permeable pores than do the filling samples with n = 0.7 and n = 0.9. The pores are relatively wide, but the consolidation ability is slightly lower. Disorderly water inrush crevices are observed. The filling samples with gradation values of n = 0.7 and n = 0.9 attain good consolidation around their pores and achieve a certain water blocking effect. The above test results show that particle migration is an important reason for the difference in penetration pores between the tested collapse column fillings.

**Figure 12:**
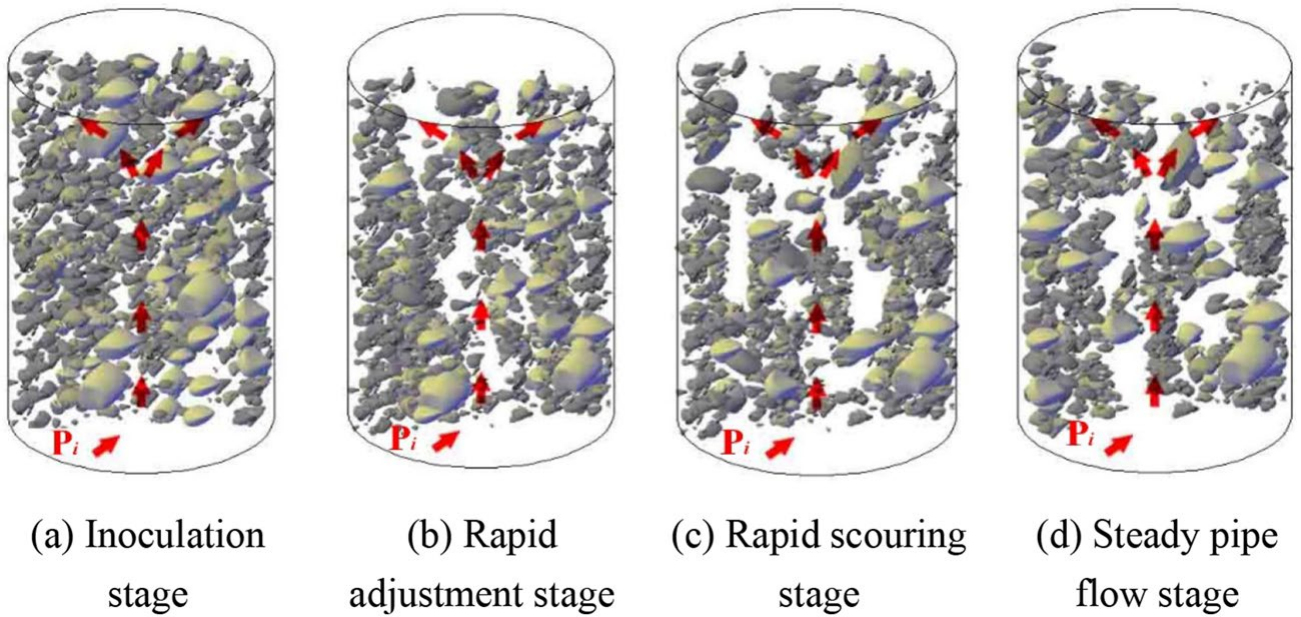
Schematic diagram of the pore penetration process of a filling sample.

According to the test settings, the pore penetration process in the filling samples of the various gradation values at the different osmotic pressure levels can be summarized into 4 stages of inoculation, rapid adjustment, rapid scouring and steady-state pipe flow, as shown in Fig. 12.

Figure 12 shows that at the early stage of infiltration, the filling samples with gradation values of n = 0.3 and n = 0.5 contained more primary fine particles. Under the action of the pore water pressure, the fine particles in the filling samples rapidly adjusted within a short time, and (a) fine particles were quickly lost with the water flow after incubation (b). When the osmotic water pressure was continuously increased, fewer fine particles remained that could migrate, the water blocking capacity between particles was gradually weakened, and the filling particles entered the rapid adjustment stage (c). Most of the particles that could migrate mainly included secondary fine particles newly produced by water–rock interactions. When the migrating particles entered a certain stage, a stable water channel was formed, and the water flow was subsequently transformed into relatively stable pipe flow (d). However, the case of the filling samples with intermediate gradation values n = 0.7 and n = 0.9 exhibited the complete opposite behaviour because these samples contained more large particles. Therefore, small particles effectively filled pores after compression, which effectively reduced the seepage channel within the samples.

## Conclusion

1. Among the 5 levels of penetration loading, there is a peak compaction load for the samples at each level, namely, approximately 35.3 N. Large-scale load fluctuation generally occurs at the middle and late stages of the 10-kN level. The performance of the samples with gradation values of n = 0.3 and n = 0.5 is very obvious. At the beginning of loading, the samples at all levels attain a certain hydraulic bearing capacity. The samples with gradation values of n = 0.3 and n = 0.5 produce fine particles that easily migrate, the interior of the filling may exhibit collapse and plugging behaviours, and the load fluctuation characteristics are obvious. The hydraulic bearing capacity of the samples with gradation values of n = 0.7 and n = 0.9 is relatively good, and storage and migration behaviours of fine particles occur between the pores.
2. During the entire pore infiltration process, the mass of migrating particles m decreases with increasing gradation value n, and the total mass of migrating particles m is negatively correlated with the load F. An exponential function can be used to better fit the porosity φ and load F, and the porosity decreases with increasing load.
3. After infiltration, the remaining rock skeleton samples in the cylinder generally show a trend with a rapid decrease first, then a slow increase and finally a decrease. The particle size range of 8–10 mm is regarded as the boundary, and there is a small amount of aggregates with a particle size larger than 20 mm. The relationship between the change in seepage velocity and time is very similar at all load levels. At the first stage of the 10-kN load level, there is a sudden change point, and the water flow fluctuates greatly. The performance of the samples with gradation values of n = 0.3 and n = 0.5 is very obvious. Maximum seepage velocities of 0.0193 and 0.0176 m2/s, respectively, are observed.
4. Migrating particles are an important reason for the difference in penetration pores between the different collapse column fillings. The gradation value n of the sample exhibits a good correlation with the average maximum area S of the bottom failure area and top water inrush channel. The maximum increase in the bottom failure area and the maximum area increase in the top water inrush channel indicate opposite trends, and there is a certain intersection point between the damage area and water inrush channel, which mostly occurs among the filling samples with gradation values of n = 0.7 and n = 0.9. The permeable pores in the graded samples can be divided into three categories, namely, erosion collapse pores, water inrush crevices and scouring holes, while the pore permeation process in the samples undergoes four stages of inoculation, rapid adjustment, rapid scouring and steady-state pipe flow.

## Data Availability

All data generated or analysed during this study are included in this published article.
